# Preventing hypothermia in elective arthroscopic shoulder surgery patients: a protocol for a randomised controlled trial

**DOI:** 10.1186/1471-2482-12-14

**Published:** 2012-07-20

**Authors:** Jed Duff, Renatta Di Staso, Kerry-Anne Cobbe, Nicole Draper, Simon Tan, Emma Halliday, Sandy Middleton, Lawrence Lam, Kim Walker

**Affiliations:** 1 St Vincent’s Private Hospital, Victoria Street, Darlinghurst, NSW, 2010, Australia; 2 Nursing Research Institute, St Vincent’s & Mater Health Sydney-Australian Catholic University, Victoria Street, Darlinghurst, NSW, 2010, Australia; 3 National Centre for Clinical Outcomes Research (NaCCOR), Nursing and Midwifery, ACU, Australia; 4 The University of Notre Dame Australia, 60 Oxford Street, Darlinghurst, NSW, 2010, Australia

## Abstract

**Background:**

Patients having arthroscopic shoulder surgery frequently experience periods of inadvertent hypothermia. This common perioperative problem has been linked to adverse patient outcomes such as myocardial ischaemia, surgical site infection and coagulopathy. International perioperative guidelines recommend patient warming, using a forced air warming device, and the use of warmed intraoperative irrigation solutions for the prevention of hypothermia in at-risk patient groups. This trial will investigate the effect of these interventions on patients’ temperature, thermal comfort, and total recovery time.

**Method/Design:**

The trial will employ a randomised 2 x 2 factorial design. Eligible patients will be stratified by anaesthetist and block randomised into one of four groups: Group one will receive preoperative warming with a forced air warming device; group two will receive warmed intraoperative irrigation solutions; group three will receive both preoperative warming and warmed intraoperative irrigation solutions; and group four will receive neither intervention. Participants in all four groups will receive active intraoperative warming with a forced air warming device. The primary outcome measures are postoperative temperature, thermal comfort, and total recovery time. Primary outcomes will undergo a two-way analysis of variance controlling for covariants such as operating room ambient temperature and volume of intraoperative irrigation solution.

**Discussion:**

This trial is designed to confirm the effectiveness of these interventions at maintaining perioperative normothermia and to evaluate if this translates into improved patient outcomes.

**Australian New Zealand Clinical Trials Registry number:**

ACTRN12610000591055

## Background

Inadvertent perioperative hypothermia, defined as a core temperature below 36°C [1], is a common, yet widely under-acknowledged adverse clinical consequence of surgery [1-3]. Patients undergoing arthroscopic shoulder surgery are particularly at risk, with the average patient experiencing a core temperature heat loss of between 1°C and 3°C [4-6]. Three principle factors are said to contribute to this problem: Reduced metabolic heat production due to the anaesthetic; heat loss due to the cold perioperative environment and the use of large quantities of surgical irrigation solution; and impaired thermoregulation which results in a core to periphery thermal redistribution [7].

Although hypothermia is a common perioperative problem, it is not a benign one: The consequences are both physiological and psychological in nature and are far more serious than patients just ‘feeling uncomfortably cold’. Research has demonstrated a clear link between inadvertent perioperative hypothermia and serious adverse complications including myocardial ischaemia, surgical site infection, and coagulopathy [1,8-11]. A person’s temperature is also an integral component of their overall perception of well-being and research has shown that memories of thermal discomfort during the perioperative period significantly affect a patient’s surgical experience [12,13]. These physiological and psychological adverse effects can, and do, result in prolonged recovery times, lengthier hospital stays, and increased resource use which in turn translates into greater overall healthcare costs [10,11].

A number of active and passive interventions are recommended in the evidence-based guidelines for maintaining normothermia in perioperative patients [1,14]. Two relatively simple and inexpensive interventions which are not routinely used on patients undergoing shoulder arthroscopy surgery are preoperative warming using a forced air warming device and the use of warmed intraoperative irrigation solutions [7].

### Preoperative warming

The preoperative warming of patients at high risk of hypothermia, such as those having arthroscopic surgery, is recommended in evidence-based guidelines [1,14]. Warming the peripheral tissues preoperatively reduces the impact of core to periphery thermal redistribution caused by anaesthetic-induced peripheral vasodilatation [15]. Consequently, patients experience less post-induction temperature loss and recover from any loss at a faster rate intraoperatively [16-19]. A forced air warming device has been shown to be the most effective method for preoperative warming, consistently demonstrating higher core temperatures in preoperative normothermic patients compared to other warming techniques [17,19-23].

### Warmed intraoperative irrigation solutions

It is well documented that the use of room temperature irrigation solution increases the risk of inadvertent perioperative hypothermia during arthroscopic surgery. A systematic review of 13 randomised controlled trials including 686 patients showed that room-temperature irrigation fluid caused a greater drop in core body temperature and more episodes of hypothermia in patients, than warmed irrigation fluid [4]. There is a significant correlation between the volume of room temperature irrigation solution used and a patients’ mean postoperative temperature [6,24]. The use of warmed solution for intraoperative irrigation during arthroscopic surgery has been recommended as a method for preventing perioperative hypothermia [1,4,14].

There is clear evidence that these two interventions assist in the maintenance of perioperative normothermia but there is now a call for robust well designed research to demonstrate improved clinical outcomes associated with their use [1]. This trial will study the effects of these warming interventions on outcomes of particular interest to perioperative nurses, namely, post-operative temperature, thermal comfort, and total recovery time.

### Purpose

To investigate the effect of preoperative forced air warming and warmed intraoperative irrigation solution, alone and in combination, on postoperative temperature, thermal comfort, and total recovery time in adult patients undergoing elective arthroscopic shoulder surgery.

## Method

### Trial design

The trial will employ a randomised 2 x 2 factorial design. An equal ratio of participants will be allocated to each group. This design will enable the study of each intervention on the outcome variable, as well as the effects of interactions between interventions on the outcome variable.

### Setting

The trial will be conducted in the day surgery unit of a private hospital in Sydney, Australia.

### Eligibility criteria

Participants will be deemed eligible for the trial if they are over the age of 18 years and are scheduled for elective arthroscopic shoulder surgery. They must be classified as American Society of Anaesthesiologists grade I-III (see Table 1) and have a body mass index between 18.5 and 40. Patients will be excluded if they have a preoperative temperature above 37.5°C or if they are unable to speak or understand English.

**Table 1:** American Society of Anaesthesiologists grading

**ASA**	**Description**
I	Healthy individual with no systemic disease
II	Mild systemic disease not limiting activity
III	Severe systemic disease that limits activity but is not incapacitating
IV	Incapacitating systemic disease which is constantly life-threatening
V	Moribund, not expected to survive 24 hours with or without surgery

### Interventions

Participants will be allocated to one of four groups (see Table 2): Group one will receive preoperative warming with a forced air warming device; group two will receive warmed intraoperative irrigation solutions; group three will receive both preoperative warming and warmed intraoperative irrigation solutions; and group four will comprise the control group receiving neither intervention.

**Table 2:** Interventions allocated to each study group

	**Preoperative warming**	**No preoperative warming**
**Warmed irrigation solutions**	Group 1	Group 2
**No warmed irrigation solutions**	Group 3	Group 4

#### Preoperative forced air warming

Those allocated to group 1 and 3 will be changed into a hospital gown and seated in a recliner chair in the preoperative holding area. A commercial warming blanket will be applied to approximately 50% of their anterior body surface and a hospital sheet placed on top. They will then receive 45 minutes of preoperative forced air warming (Bair Hugger® model number 775) with the device temperature set at 43°C. Participants will be monitored for sweating, flushing and thermal discomfort and the device temperature titrated accordingly.

#### Warmed intraoperative irrigation solution

Patients allocated to group 1 and 2 will have their intraoperative irrigation solutions warmed to 37°C [24] in a thermostatically controlled warming cabinet. The warming cabinet will be located in the operating room in which the solutions will remain until they are required. A process of random quality checks will be instigated to confirm the temperature of the irrigation fluid.

#### Usual care

Participants in group four will receive ‘usual care’ only. This does not include preoperative warming or the use of warmed intraoperative irrigation solutions. All four groups will receive active intraoperative warming with a forced air warming device (Bair Hugger® model number 775) for the duration of their surgery.

### Outcomes

**Figure 1:**
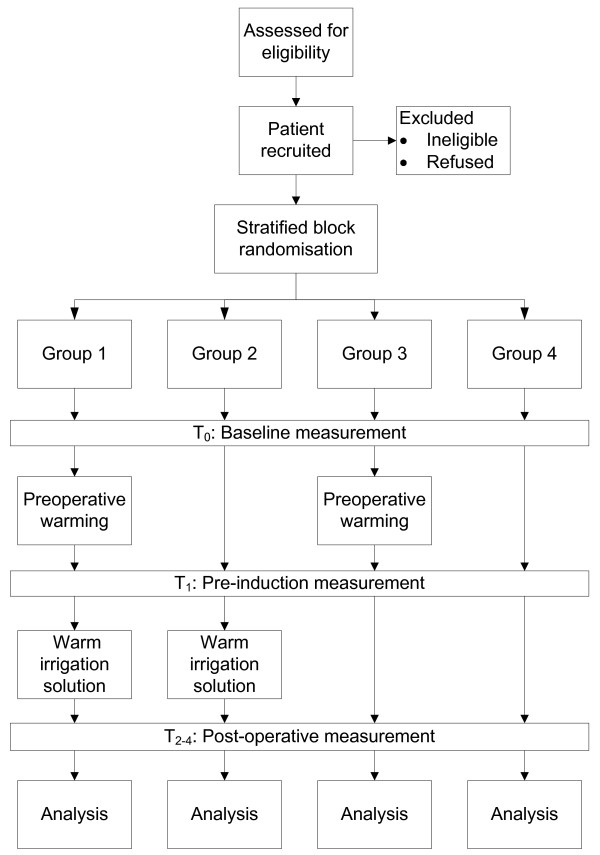
Study flow diagram.

The primary outcome measure in this trial is postoperative temperature. The secondary outcomes of interest are thermal comfort and total recovery time. Baseline data on patient temperature and thermal comfort will be collected on arrival into the department (T_0_). Follow-up measures will be collected at four time points; immediately prior to induction (T_1_), on arrival into the recovery unit (T_2_), 20 minutes after arrival into the recovery unit (T_3_) and immediately prior to discharge from the recovery unit (T_4_) (see Figure 1).

#### Temperature

Temperature will be measured by nursing staff using a dedicated tympanic thermometer (Welch Allyn Braun Thermoscan® PRO 4000). This device has been shown to be reliable when tested against readings from a pulmonary artery catheter (the ‘gold standard’ in core temperature assessment) and is deemed more accurate than other similar devices [25]. The thermometer will be calibrated by the clinical engineering department (as per the manufacturer’s instructions). All nursing and medical staff will receive instruction in its use prior to the trial commencing.

#### Thermal comfort

Thermal comfort will be measured on a 10 point self-reported thermal comfort scale. This type of numeric rating scale has been used effectively in other studies on thermal comfort [26,27]. Participants will be asked, using a standardised script, to score how comfortable they are with their body temperature on a scale from 0 to 10 with zero being very comfortable (neither too hot nor cold) and 10 being very uncomfortable (too hot or cold).

#### Total recovery time

Total recovery time will be calculated from the patient’s arrival into the recovery unit until the time they are deemed fit for discharge from the recovery unit by the recovery nurse. Fitness for discharge will be assessed using a standardised post anaesthetic discharge scoring tool (see Table 3) [28].

**Table 3:** Aldrete and Kroulik modified post-anaesthetic recovery score. A total score >8 indicates recovery from anaesthetic

**Variable**		**Score**
Consciousness	Fully awake and oriented (name, place, date)	2
	Awake when called	1
	Not responding	0
Activity	Moves all four extremities on command	2
	Moves two extremities	1
	Unable to move extremities	0
Respiration	Breathes deeply and coughs freely	2
	Dyspnoea, limited breathing, or tachypnoea	1
	Apnoeic	0
Circulation	BP ±20% of pre-anaesthetic level	2
	BP ±20%–50% of pre-anaesthetic level	1
	BP ±50% of pre-anaesthetic level	0
Peripheral oxygen saturation	>92% on room air	2
	>92% with oxygen	1
	<92% with oxygen	0

### Sample size

The trial has been powered to detect a 0.05°C (SD 0.5) difference in temperature between the four study groups. This was deemed a clinically significant difference based on the researchers experience and previous published studies [12,24]. Based on this number, and given the trial’s design, a total sample size of 120 participants is required to power the trial at 80% with a significance level of 5%.

### Interim analysis

An interim analysis of efficacy will be performed when 75% of participants have been enrolled in the trial. The level of significance will maintain an overall P value of 0.05 and be calculated according to the O’Brien-Fleming stopping boundaries [29].

### Randomisation

A statistician, with no clinical involvement in the trial, will computer-generate a stratified (by anaesthetist) block randomised sequence. This list will remain concealed from the trial coordinator at all times. When the trial coordinator has assessed and enrolled a participant, she will telephone an independent person to obtain the treatment allocation. She will then inform the appropriate nursing and medical staff who will deliver the intervention(s). The trial coordinator will not collect outcome data, deliver the intervention(s), or provide patient care.

### Blinding

Outcome measures will be collected by recovery unit nursing staff who are blinded to the participants’ treatment allocation. The two interventions will be delivered by separate groups of perioperative staff (preoperative and intraoperative staff) and each group will be blinded to the treatment delivered by the other. The trial will adhere to procedures to maintain separation between the recovery unit nursing staff who will record the outcome data and the preoperative and intraoperative nursing and medical staff who will deliver the intervention(s). Due to the difficulty in blinding participants to the preoperative warming intervention, they will be blinded to the trial hypothesis and design [30].

### Statistical analysis

Intention-to-treat analysis will be applied. Data will be analysed according to the 2x2 randomised factorial study design. The two-way analysis of covariance (two-way ANCOVA) will be used for the primary and secondary outcome measures of temperature, thermal comfort and total recovery time. The two-way ANCOVA model will also include operating room ambient temperature, volume of irrigation solution, length of surgery, blood loss and other covariates identified in the bivariate analyses. The analysis will be adjusted for baseline temperature and thermal comfort. Pair-wise comparison between groups will be conducted based on the results obtained from the two-way ANCOVA.

### Ethical considerations

The project has been approved by the hospital’s human research ethics committee. Informed consent will be obtained from all participants.

## Discussion

This trial is the first to rigorously evaluate the effect of preoperative warming and the use of warmed intraoperative irrigation solution on outcomes of particular interest to perioperative nurses, namely thermal comfort, and total recovery time. The factorial design of the trial enables a head-to-head comparison of the individual and cumulative effects of these two interventions which should provide valuable evidence to inform perioperative clinical practice.

## Competing interests

The authors declare that they have no competing interests.

## Authors’ contributions

All authors have contributed to trial design and have reviewed and approved the final manuscript. JD wrote the first draft of the manuscript. RD and KC developed the data collection tool and data collection process. LL provided the data analysis plan and sample size calculation.

## Pre-publication history

The pre-publication history for this paper can be accessed here:

http://www.biomedcentral.com/1471-2482/12/14/prepub
